# Quadratic Split Quaternion Polynomials: Factorization and Geometry

**DOI:** 10.1007/s00006-019-1037-1

**Published:** 2019-12-17

**Authors:** Daniel F. Scharler, Johannes Siegele, Hans-Peter Schröcker

**Affiliations:** Department of Basic Sciences in Engineering Sciences, University of Innsbruck, Techikerstr. 13, 6020 Innsbruck, Austria

**Keywords:** Skew polynomial ring, Null quadric, Clifford translation, Left/right ruling, Zero divisor, Projective geometry, Non-Euclidean geometry, Primary 12D05, Secondary 12D05, 16S36, 51M09, 51M10, 70B10

## Abstract

We investigate factorizability of a quadratic split quaternion polynomial. In addition to inequality conditions for existence of such factorization, we provide lucid geometric interpretations in the projective space over the split quaternions.

## Introduction

Quaternions and dual quaternions provide compact and simple parametrizations for the groups $\mathrm {SO}(3)$, $\mathrm {SE}(2)$ and $\mathrm {SE}(3)$. This accounts for their importance in fields such as kinematics, robotics and mechanism science. In this context, polynomials over quaternion rings in one indeterminate can be used to parameterize rational motions. Factorization of polynomials corresponds to the decomposition of a rational motion into rational motions of lower degree. Since linear factors generically describe rotational motions, factorizations with linear factors give rise to a sequence of revolute joints from which mechanisms can be constructed [5].

In recent years, the theory of quaternion polynomial factorization [4, 13] has been extended to the dual quaternion case and numerous applications have been found [9–11]. The main difficulty in comparison with the purely quaternion theory is the presence of zero divisors. As of today our general understanding of dual quaternion factorization is quite profound but some questions still remain. Most notably, a complete characterization of all polynomials that admit factorizations with only linear factors and algorithms for computing them are still missing. Both exist for “dense” classes of dual quaternion polynomials [11].

A first step in research on factorizability and factorization algorithms of polynomials should be the investigation of quadratic polynomials. This has been done for quaternions in [6] and for split quaternions in [2]. Results for generalized quaternions, including split quaternions, can also be found in [1]. The generic case is subsumed in a generic factorization theory as in [11, 12] while special cases still allow a complete discussion.

In this article, we consider quadratic left polynomials over the split quaternions. Factorization results for these polynomials are among the topics of [1, 2]. We present different characterizations, tailored towards later geometric interpretation of factorizability, based on the geometry of split quaternions. It is much clearer than the inequality criteria with their numerous case distinctions that had been known so far (c. f. Theorem 3.13, Corollarys 3.17 and 3.18). Moreover, we also use our criteria for covering polynomials with non-invertible leading coefficient which hitherto have not been dealt with.

Factorization of quadratic polynomials over the split quaternions is interesting from a purely algebraic viewpoint because, like dual quaternions but unlike ordinary quaternions, the ring of split quaternions contains zero divisors. Their structure is more involved than in the dual quaternion case but, nonetheless, allows a reasonably simple computational treatment and a nice geometric interpretation. It is also relevant in hyperbolic kinematics [12] and isomorphic to the fundamental algebra of real $2 \times 2$ matrices.

The remainder of this article is structured as follows: Following a presentation of basic results on split quaternions and their geometry in Sect. 2, our main results are given in Sect. 3. We first derive our own inequality conditions and their geometric interpretation for the cases of dependent and independent coefficients under the assumption that the norm polynomial does not vanish in Sects. 3.1 and 3.2. A geometric interpretation of these cases is given in Sect. 3.3. The remaining case of vanishing norm polynomial is the topic of the concluding Sect. 3.4.

## Preliminaries

### Split Quaternions and Split Quaternion Polynomials

The algebra of split quaternions, denoted by $\mathbb {S}$, is generated by the quaternion units $\mathbf {i}$, $\mathbf {j}$ and $\mathbf {k}$ over the real numbers $\mathbb {R}$. An element $h \in \mathbb {S}$ is given by $h = h_0 + h_1 \mathbf {i}+ h_2 \mathbf {j}+ h_3 \mathbf {k}$, where $h_0$, $h_1$, $h_2$, $h_3 \in \mathbb {R}$ are real numbers. The multiplication of split quaternions is defined by the relations

$$\begin{aligned} \mathbf {i}^2 = - \mathbf {j}^2 = - \mathbf {k}^2 = -\mathbf {i}\mathbf {j}\mathbf {k}= -1. \end{aligned}$$

From this, the complete multiplication table may be inferred and one finds that the basis elements anti-commute, e.g. $\mathbf {i}\mathbf {j}= -\mathbf {j}\mathbf {i}$. The split quaternion *conjugate* to $h = h_0 + h_1 \mathbf {i}+ h_2 \mathbf {j}+ h_3 \mathbf {k}$ is defined as ${h}^* :=h_0 - h_1 \mathbf {i}- h_2 \mathbf {j}- h_3 \mathbf {k}$. Conjugation of split quaternions $h \mapsto {h}^*$ is an anti-automorphism, i.e. ${(hg)}^* = {g}^* {h}^*$ for *h*, $g \in \mathbb {S}$. The split quaternion norm is defined by $h {h}^* = {h}^* h = h_0^2 + h_1^2 - h_2^2 - h_3^2 \in \mathbb {R}$. A split quaternion *h* is invertible if and only if $h{h}^* \ne 0$ in which case $h^{-1} = (h {h}^*)^{-1} {h}^*$. The *scalar* or *real part* of $h \in \mathbb {S}$ is ${{\,\mathrm{Re}\,}}(h) :=\frac{1}{2}(h + {h}^*) = h_0$, the *vector* or *imaginary part* is ${{\,\mathrm{Im}\,}}(h) :=\frac{1}{2}(h - {h}^*) = h_1\mathbf {i}+ h_2\mathbf {j}+ h_3\mathbf {k}$. The split quaternion *h* is called *vectorial* if ${{\,\mathrm{Re}\,}}(h) = 0$.

By $\mathbb {S}[t]$ we denote the ring of polynomials in one indeterminate *t* with split quaternion coefficients. Addition is done in the usual way; multiplication is defined by the convention that the indeterminate *t* commutes with all coefficients in $\mathbb {S}$. This is motivated by applications in hyperbolic kinematics [12] where *t* serves as a real motion parameter that, indeed, is in the center of $\mathbb {S}$. Consider a left polynomial $P = \sum _{\ell = 0}^n p_\ell t ^\ell \in \mathbb {S}[t]$ (coefficients are written to the left hand side of the indeterminate *t*). The *conjugate polynomial* ${P}^* :=\sum _{\ell = 0}^n {p}^*_{\ell } t^\ell$ is obtained by conjugation of the coefficients. Hence, the norm polynomial $P {P}^* = {P}^* P \in \mathbb {R}[t]$ is real. The *evaluation* of *P* at $h \in \mathbb {S}$ is defined by $P(h) :=\sum _{\ell = 0}^n p_\ell h ^\ell$. One calls it a *right evaluation* because the variable *t* is written to the right hand side of the coefficients and then substituted by *h*. To illustrate the substantial difference to the left evaluation (of right polynomials) where the variable *t* is written to the left hand side of the coefficients, consider the polynomial $h_1 t = t h_1 \in \mathbb {S}[t]$ (*t* commutes with $h_1$) and a split quaternion $h_2 \in \mathbb {S}$. Right evaluation of $h_1 t$ at $h_2$ yields $h_1 h_2$ whereas left evaluation of $t h_1$ yields $h_2 h_1$. The results are different unless $h_1$ and $h_2$ commute.

Due to non-commutativity of split quaternion multiplication we have to differ between right and left factors and zeros of a polynomial as well. Consider two split quaternion polynomials *P*, $F \in \mathbb {S}[t]$. We call *F* a right factor of *P* if there exists a polynomial $Q \in \mathbb {S}[t]$ such that $P = Q F$. A right zero *h* of a left polynomial *P* is defined by the property that the right evaluation of *P* at *h* vanishes. Left factors and left zeros are defined analogously. In this paper we mainly deal with left polynomials, right evaluation, right factors and right zeros but often simply speak of polynomials, evaluation, factors and zeros, respectively. Of course, there exists a symmetric theory on right polynomials and left evaluation, factors and zeros.

### Geometry of Split Quaternions

In this section we take a look at the geometry of split quaternions which, as we shall see, is closely related to factorizability of split quaternion polynomials. In particular, the symmetric bilinear form

$$\begin{aligned} q:\mathbb {S}\times \mathbb {S}\rightarrow \mathbb {R}, \quad (h,g) \mapsto \frac{1}{2}(h {g}^* + g {h}^*) \end{aligned}$$

will play a vital role. Since it is of signature (2, 2), the real four-dimensional vector-space $\mathbb {S}$ together with *q* is a *pseudo-Euclidean space.* Its *null cone* consists of all split quaternions *h* that satisfy $q(h,h) = 0$. Because of $q(h,h) = h{h}^*$, these are precisely the split quaternions of vanishing norm.

Some aspects of polynomial factorization over split quaternions have a geometric interpretation in this pseudo-Euclidean space while others are of projective nature. Hence, we also consider the projective space $\mathbb {P}(\mathbb {S})$ over $\mathbb {S}$. Any vector $h \in \mathbb {S}{\setminus }\{0\}$ represents a point in $\mathbb {P}(\mathbb {S})$ which we denote by [*h*]. Projective span is denoted by the symbol “$\vee$”, i.e., the straight line spanned by two different points $[h_1]$, $[h_2] \in \mathbb {P}(\mathbb {S})$ is $[h_1] \vee [h_2]$.

#### Definition 2.1

The quadric $\mathcal {N}$ in $\mathbb {P}(\mathbb {S})$ represented by the symmetric bilinear form *q* is called the *null quadric*. Lines contained in $\mathcal {N}$ are called *null lines*.

Because the signature of *q* is (2, 2), the null quadric $\mathcal {N}$ is of hyperbolic type and two families of null lines do exist. We illustrate the importance of $\mathcal {N}$ to the algebra of split quaternions by a few results which we will need later.

#### Lemma 2.2

([12]) Let $r_0$, $r_1 \in \mathbb {S}$ be two linearly independent split quaternions. The straight line $[r_0] \vee [r_1]$ is a null line if and only if the polynomial $R = r_1 t + r_0$ satisfies $R {R}^* = 0$.

#### Proof

The line $[r_0] \vee [r_1]$ is contained in $\mathcal {N}$ if and only if $q(r_0,r_0) = q(r_0,r_1) = q(r_1,r_1) = 0$ [3, Lemma 6.3.3]. On the other hand we have

$$\begin{aligned} R {R}^* = r_1 {r}^*_1 t^2 + (r_0 {r}^*_1 + r_1 {r}^*_0) t + r_0 {r}^*_0 = q(r_1,r_1) t^2 + 2 q(r_0, r_1) t + q(r_0, r_0) \end{aligned}$$

and the two statements are equivalent. $\square$

The two families of null lines can be distinguished by algebraic properties of split quaternions.

#### Theorem 2.3

If [*h*] is a point of $\mathcal {N}$, the sets

2.1 $$\begin{aligned} {\mathcal {L}} :=\{ [r] \mid r{h}^* = 0 \} \quad \text {and}\quad {\mathcal {R}} :=\{ [r] \mid {h}^*r = 0\} \end{aligned}$$

are the two different rulings of $\mathcal {N}$ through [*h*].

#### Proof

We observe that the system of homogeneous linear equations in the coefficients of $x \in \mathbb {S}$ resulting from $xg = 0$ (or $gx = 0$) with $g \in \mathbb {S}{\setminus }\{0\}$ has non trivial solutions if and only if $[g] \in \mathcal {N}$. In this case, the vector-space of solutions is of dimension two. This already implies that ${\mathcal {L}}$ and ${\mathcal {R}}$ are straight lines.

Consider $[r] \in {\mathcal {L}}$. We have $2 \, q(h,r) = h{r}^* + r{h}^* = {(r{h}^*)}^* + r{h}^* = 0$ and ${\mathcal {L}}$ lies in the tangent plane of $\mathcal {N}$ in [*h*]. We choose $p \in \mathbb {S}$ such that $ph \ne 0$ and $[ph] \ne [h]$. This is possible since the solutions set of $xh = 0$ is a vector-space of dimension two and the set of all real multiples of *h* is a vector-space of dimension one. From $(ph){(ph)}^* = p(h{h}^*){p}^* = 0$ we infer that the point [*ph*] lies on $\mathcal {N}$. Moreover, it is contained in ${\mathcal {L}}$ because of $(ph){h}^* = p(h{h}^*) = 0$. Obviously, *h* is contained in ${\mathcal {L}}$ as well. Summing up, ${\mathcal {L}}$ is in the tangent plane of $\mathcal {N}$ in [*h*] and contains two distinct points [*h*], $[ph] \in \mathcal {N}$. Hence, it is a null line through *h*.

Similar arguments demonstrate that ${\mathcal {R}}$ is a null line through [*h*] as well. The two rulings ${\mathcal {L}}$ and ${\mathcal {R}}$ cannot be the same as the equation systems $hx = 0$ and $xh = 0$ are not equivalent. $\square$

#### Definition 2.4

The ruling ${\mathcal {L}}$ in Theorem 2.3 is called a *left ruling* of $\mathcal {N}$, the ruling ${\mathcal {R}}$ is called a *right ruling.*

#### Corollary 2.5

Consider two points $[h] \in \mathcal {N}$ and $[p] \in \mathbb {P}(\mathbb {S})$.

- If *p* is such that $ph \ne 0$ and $[ph] \ne [h]$, then $[h] \vee [ph]$ is a left ruling of $\mathcal {N}$.
- If *p* is such that $hp \ne 0$ and $[hp] \ne [h]$, then $[h] \vee [hp]$ is a right ruling of $\mathcal {N}$.

#### Proof

Consider $p \in \mathbb {S}$ with $ph \ne 0$ and $[ph] \ne [h]$. As argued in the proof of Theorem 2.3, such a choice is possible. We have $h{h}^* = 0$ and $ph{h}^* = 0$. This implies that [*h*] and [*ph*] lie on the same left ruling by Theorem 2.3. Therefore we have two different points on a left ruling and the span $[h] \vee [ph]$ of these points is the ruling itself. The second statement is similar. $\square$

#### Remark 2.6

For fixed $p \in \mathbb {S}{\setminus }\{0\}$ the maps $[x] \mapsto [px]$ and $[x] \mapsto [xp]$ are the well-known Clifford left and right translations of non-Euclidean geometry.

#### Corollary 2.7

Given split quaternions *h*, $g \in \mathbb {S}{\setminus }\{0\}$ such that $[h] \vee [g]$ is a left (right) ruling of $\mathcal {N}$, there exists an affine two-plane of split quaternions *p* such that $g = ph$ ($g = hp$).

#### Proof

The split quaternion equation $g = xh$ results in a system of in-homogeneous linear equations for the coefficients of *x*. We already argued in our proof of Theorem 2.3 that the solution space of the corresponding system of homogeneous equations is of dimension two. $\square$

Corollary 2.7 is a pure existence result. The next theorem provides a parametrization of the affine two-plane in Corollary 2.7. The main idea of the proof is to derive properties of a split quaternion $p = p_0 + p_1 \mathbf {i}+ p_2 \mathbf {j}+ p_3 \mathbf {k}$ by finding relations between its “positive” part $p_0 + p_1 \mathbf {i}$ and its “negative” part $p_2 \mathbf {j}+ p_3 \mathbf {k}$. These terms are motivated by the sign of their respective norms.

#### Theorem 2.8

Suppose that $h = h_0 + h_1 \mathbf {i}+ h_2 \mathbf {j}+ h_3 \mathbf {k}\in \mathbb {S}\setminus \{ 0 \}$ and $g = g_0 + g_1 \mathbf {i}+ g_2 \mathbf {j}+ g_3 \mathbf {k}\in \mathbb {S}{\setminus }\{ 0 \}$ are as in Corollary 2.7. The affine two-plane consisting of all split quaternions $x \in \mathbb {S}$ solving the equation $g = xh$ can be parameterized by $u + \lambda {h}^* + \mu \mathbf {i}{h}^*$, where $u = (g_0 + g_1 \mathbf {i}) (h_0 + h_1 \mathbf {i})^{-1}$ and $\lambda$, $\mu \in \mathbb {R}$. (The same statement holds for $g = hx$ with $u + \lambda {h}^* + \mu {h}^* \mathbf {i}$ where $u = (h_0 + h_1 \mathbf {i})^{-1} (g_0 + g_1 \mathbf {i})$ and $\lambda$, $\mu \in \mathbb {R}$.)

#### Proof

Regarding the system of linear equations arising from $xh = g$ we have to show the following:

- *u* solves $$xh = g$$,
- ${h}^*$ and $\mathbf {i}{h}^*$ solve the corresponding homogeneous system $xh = 0$, and
- ${h}^*$ and $\mathbf {i}{h}^*$ are linearly independent.

(Note that we already know that the solution space is of dimension two.)

The condition that $[h] \vee [g]$ is a null line yields

2.2 $$\begin{aligned} h {h}^* = g {g}^* = h {g}^* + g {h}^* = 0. \end{aligned}$$

Moreover, it is a left ruling of $\mathcal {N}$ and therefore

2.3 $$\begin{aligned} h {g}^* = g {h}^* = 0 \end{aligned}$$

by Eq. (2.1). From Eq. (2.2) we obtain

2.4 $$\begin{aligned} 0 = h {h}^* = (h_0 + h_1 \mathbf {i}) {(h_0 + h_1 \mathbf {i})}^* + (h_2 \mathbf {j}+ h_3 \mathbf {k}) {(h_2 \mathbf {j}+ h_3 \mathbf {k})}^* \end{aligned}$$

and, because $h \ne 0$, the norms of $h_0 + h_1 \mathbf {i}$ and $h_2 \mathbf {j}+ h_3 \mathbf {k}$ are different from zero. Hence, $h_0 + h_1 \mathbf {i}$ and $h_2 \mathbf {j}+ h_3 \mathbf {k}$ are both invertible and $u = (g_0 + g_1 \mathbf {i}) (h_0 + h_1 \mathbf {i})^{-1}$ is well defined. We have

2.5 $$\begin{aligned} \begin{aligned} u h&= (g_0 + g_1 \mathbf {i}) (h_0 + h_1 \mathbf {i})^{-1} (h_0 + h_1 \mathbf {i}+ h_2 \mathbf {j}+ h_3 \mathbf {k}) \\&= g_0 + g_1 \mathbf {i}+ (g_0 + g_1 \mathbf {i}) (h_0 + h_1 \mathbf {i})^{-1} (h_2 \mathbf {j}+ h_3 \mathbf {k}) \\&= g_0 + g_1 \mathbf {i}+ \frac{1}{(h_0 + h_1 \mathbf {i}) {(h_0 + h_1 \mathbf {i})}^*} (g_0 + g_1 \mathbf {i}) {(h_0 + h_1 \mathbf {i})}^* (h_2 \mathbf {j}+ h_3 \mathbf {k}). \end{aligned} \end{aligned}$$

Equation (2.3) yields

$$\begin{aligned} 0 = g {h}^*&= (g_0 + g_1 \mathbf {i}) {(h_0 + h_1 \mathbf {i})}^* + (g_2 \mathbf {j}+ g_3 \mathbf {k}) {(h_2 \mathbf {j}+ h_3 \mathbf {k})}^*\\&\quad +(g_0 + g_1 \mathbf {i}) {(h_2 \mathbf {j}+ h_3 \mathbf {k})}^* + (g_2 \mathbf {j}+ g_3 \mathbf {k}) {(h_0 + h_1 \mathbf {i})}^* \end{aligned}$$

where the first two terms form the positive part (they are in the span of 1 and $\mathbf {i}$) while the two trailing terms form the negative part (they are in the span of $\mathbf {j}$ and $\mathbf {k}$). Positive and negative parts both have to vanish whence

2.6 $$\begin{aligned} \begin{aligned} 0 =&(g_0 + g_1 \mathbf {i}) {(h_0 + h_1 \mathbf {i})}^* + (g_2 \mathbf {j}+ g_3 \mathbf {k}) {(h_2 \mathbf {j}+ h_3 \mathbf {k})}^* \\ \Leftrightarrow&\, (g_0 + g_1 \mathbf {i}) {(h_0 + h_1 \mathbf {i})}^* (h_2 \mathbf {j}+ h_3 \mathbf {k}) = -(g_2 \mathbf {j}+ g_3 \mathbf {k}) {(h_2 \mathbf {j}+ h_3 \mathbf {k})}^* (h_2 \mathbf {j}+ h_3 \mathbf {k}) \end{aligned} \end{aligned}$$

Substituting Eqs. (2.6) into (2.5) we obtain via (2.4)

$$\begin{aligned}&u h = g_0 + g_1 \mathbf {i}- \frac{(h_2 \mathbf {j}+ h_3 \mathbf {k}) {(h_2 \mathbf {j}+ h_3 \mathbf {k})}^*}{(h_0 + h_1 \mathbf {i}) {(h_0 + h_1 \mathbf {i})}^*} (g_2 \mathbf {j}+ g_3 \mathbf {k})\\&= g_0 + g_1 \mathbf {i}+ g_2 \mathbf {j}+ g_3 \mathbf {k}= g \end{aligned}$$

and *u*, indeed, solves $xh = g$.

The split quaternions ${h}^* = h_0 - h_1\mathbf {i}- h_2\mathbf {j}- h_3\mathbf {k}$ and $\mathbf {i}{h}^* = h_1 + h_0\mathbf {i}+ h_3\mathbf {j}- h_2\mathbf {k}$ obviously solve the homogeneous system $xh = 0$. It remains to be shown that they are linearly independent: The positive parts are linearly dependent if and only if $h_0 = h_1 = 0$, the negative parts are linearly dependent if and only if $h_2 = h_3 = 0$. Both conditions cannot be fulfilled as $h \ne 0$. $\square$

Note that the statements of Corollary 2.7 and Theorem 2.8 hold true even if [*g*] and [*h*] do not span a line but coincide.

## Factorization Results

In this section, we investigate factorizability of quadratic split quaternion polynomials. Consider a quadratic polynomial

3.1 $$\begin{aligned} P = a t^2 + b t + c \in \mathbb {S}[t], \end{aligned}$$

where $a = a_0 + a_1 \mathbf {i}+ a_2 \mathbf {j}+ a_3 \mathbf {k}$, $b = b_0 + b_1 \mathbf {i}+ b_2 \mathbf {j}+ b_3 \mathbf {k}$, $c = c_0 + c_1 \mathbf {i}+ c_2 \mathbf {j}+ c_3 \mathbf {k}$ are split quaternions. We say that *P* *admits a factorization,* if there exist split quaternions $h_1$, $h_2$ such that

$$\begin{aligned} P = a(t - h_1)(t - h_2). \end{aligned}$$

For the time being (until Sect. 3.4) we assume that the leading coefficient *a* is invertible. In this case, we may further assume that *P* is monic because we may easily construct all factorization of *P* from factorizations of the monic polynomial $a^{-1}P$. Finally, we apply the parameter transformation $t \mapsto t - \frac{b_0}{2}$ whence $b_0 = 0$. To summarize, we investigate the factorizations

$$\begin{aligned} P = t^2 + bt + c = (t-h_1)(t-h_2) \end{aligned}$$

where *b*, *c*, $h_1$, $h_2 \in \mathbb {S}$ and ${{\,\mathrm{Re}\,}}b = 0$ (or, equivalently, $b + {b}^* = 0$).

A fundamental result (for example [8, Theorem 2]) relates factorizations to right zeros:

### Lemma 3.1

The split quaternion $h_2$ is a right zero of the (not necessarily monic) polynomial $P \in \mathbb {S}[t]$ if and only if $t - h_2$ is a right factor of *P*.

Once a right factor $t - h_2$ of a quadratic polynomial is found, a left factor $t - h_1$ can be computed by left polynomial division. Thus finding factorizations is essentially equivalent to finding right zeros and all results on right zeros of [1, 2] are of relevance to us. Nonetheless, we continue by developing our own criteria that are related to a well-known procedure [5, 8] for computing a factorization of a generic quadratic polynomial *P*:

- Pick a monic quadratic factor $M \in \mathbb {R}[t]$ of the norm polynomial $P{P}^*$.
- Compute the remainder polynomial *R* of *P* when dividing by *M*. Since *P* and *M* are monic we have $P = M + R$. Moreover, $\deg R \le 1$.
- If $R {R}^* \ne 0$, then *R* has a unique zero $h_2 \in \mathbb {S}$. The linear split quaternion polynomial $t - h_2$ is not only a factor of *R*, but also of *M* and therefore a factor of *P*.
- Right division of *P* by $t - h_2$ yields the factorization $P = (t - h_1) (t - h_2)$ with $h_1$, $h_2 \in \mathbb {S}$.

We refer to above construction as *generic factorization algorithm*. It is sufficient unless $R {R}^* = 0$. In this case the remainder polynomial *R* might not have a zero at all. If it has a zero, it already has infinitely many zeros but it is not guaranteed that they lead to right factors. In this sense, factorization of split quaternion polynomials is more interesting than factorization of polynomials over the division ring of ordinary (Hamiltonian) quaternions.

The goal of this section is to provide necessary and sufficient criteria for factorizability of all monic quadratic split quaternion polynomials $P = t^2 + bt + c$. In doing so, we consider the following sub-cases:

- *b*, $$c \in \mathbb {R}$$ $$\leadsto $$ Corollary 3.3, Lemma 3.2
- $$b \in \mathbb {R}$$ and $$c \in \mathbb {S}$$ $$\leadsto $$ Theorem 3.5
- *b*, $c \in \mathbb {S}$ and 1, *b*, *c* linearly dependent $\leadsto$ Theorem 3.6
- *b*, $c \in \mathbb {S}$ and 1, *b*, *c* linearly independent $\leadsto$ Theorem 3.7

Our structure differs from [1] but we can draw direct connections to some results. Lemma 3.2 is related to [1, Theorem 2.4.1], [1, Theorem 2.2] provides a formula to compute the roots of a polynomial given that the linear coefficient is not invertible. Although the condition for the formula to yield all roots is not met, it can be used to obtain our result in Theorem 3.5. The combination of [1, Theorem 2.2] and [1, Theorem 2.4.2] covers the second item in our Theorem 3.6.

Similar to the structure in [2] we begin our discussion with the case that the linear coefficient of *P* is real. The combination of our Lemma 3.2 and Theorem 3.5 is equivalent to [2, Theorems 2.1 and 2.2]. Finally, Theorems 3.6 and 3.7 cover the statements in [2, Theorems 4.1 and 4.2].

Regarding the remaining results in [1] or [2] it is not so straightforward to draw direct connections, one would find a set of polynomials which need to be treated by different cases with respect to our characterization but can be covered by only one theorem in [1] or [2] and vice versa.

A split quaternion $x = x_0 + x_1 \mathbf {i}+ x_2 \mathbf {j}+ x_3 \mathbf {k}\in \mathbb {S}$ is a zero of $P = t^2 + bt + c$ with ${{\,\mathrm{Re}\,}}(b) = 0$ if and only if it solves the real system of nonlinear equations

3.2 $$\begin{aligned} \begin{aligned} x_0^2 - x_1^2 + x_2^2 + x_3^2 - b_1 x_1 + b_2 x_2 + b_3 x_3 + c_0&= 0, \\ 2 x_0 x_1 + b_1 x_0 + b_3 x_2 - b_2 x_3 + c_1&= 0, \\ 2 x_0 x_2 + b_2 x_0 + b_3 x_1 - b_1 x_3 + c_2&= 0, \\ 2 x_0 x_3 + b_3 x_0 - b_2 x_1 + b_1 x_2 + c_3&= 0. \end{aligned} \end{aligned}$$

In view of Lemma 3.1, it gives rise to a right factor $t - x$ of *P*. Above system is obtained by evaluating *P* at *x* and equating the coefficients of the quaternion units $\mathbf {i}$, $\mathbf {j}$, $\mathbf {k}$ and the real coefficient with zero. Note that we are only interested in real solutions. A priori it is not obvious that this system has a solution at all. Indeed, there exist examples with zero as well as with infinitely many solutions. Below we present necessary and sufficient conditions for solvability in all cases along with some solutions.

### Factorization of Monic Polynomials with Dependent Coefficients

To begin with, we determine the zeros of the polynomial *P* in (3.1) supposing that *P* is real. In addition to the general assumptions $a = 1$ and $b_0 = 0$ this means $b_1 = b_2 = b_3 = c_1 = c_2 = c_3 = 0$. The factorization algorithm for generic polynomials (described on Page 6) fails in this setup. However, we can directly solve the polynomial system (3.2).

#### Lemma 3.2

The polynomial $P = t^2 + c_0 \in \mathbb {S}[t]$, where $c_0 \in \mathbb {R}$, has infinitely many split quaternion zeros given by the set $\{ x \in \mathbb {S}: x_0 = 0, x{x}^* = c_0 \}$. In addition, if $c_0 \le 0$, there are two real zeros $x = \pm \sqrt{- c_0}$ which coincide for $c_0 = 0$.

#### Proof

We solve the equation system (3.2) for $x = x_0 + x_1 \mathbf {i}+ x_2 \mathbf {j}+ x_3 \mathbf {k}$ under the additional assumption that $b_1 = b_2 = b_3 = c_1 = c_2 = c_3 = 0$. If $x_0 \ne 0$, we have $x_1 = x_2 = x_3 = 0$ and $x_0^2 + c_0 = 0$. Hence, *x* is real and a real solution exists if and only if $c_0 \le 0$. If so, there are two (possibly identical) solutions $x = \pm \sqrt{- c_0}$. If $x_0 = 0$, the system simplifies to the single equation $- x_1^2 + x_2^2 + x_3^2 + c_0 = 0$. Since $- x_1^2 + x_2^2 + x_3^2 = - x {x}^*$, provided that $x_0 = 0$, the set of solutions reads as $\{ x \in \mathbb {S}:x_0 = 0, x{x}^* = c_0 \}$. It is always infinite. $\square$

Combining Lemmas 3.2 with 3.1, we can state

#### Corollary 3.3

The polynomial $P = t^2 + c_0 \in \mathbb {S}[t]$ with $c_0 \in \mathbb {R}$ admits infinitely many factorizations over $\mathbb {S}$.

The solution set $\{ x \in \mathbb {S}:x_0 = 0, x{x}^* = c_0 \}$ defines a hyperboloid of one sheet, a cone or a hyperboloid of two sheets for $c_0 < 0, c_0 = 0$ or $c_0 > 0$, respectively, in the affine space ${{\,\mathrm{Im}\,}}(\mathbb {S})$.

#### Remark 3.4

Similar results can be obtained for Hamiltonian quaternions [6]. One noteworthy difference is non-negativity of the Hamiltonian norm $x_0^2 + x_1^2 + x_2^2 + x_3^2$, whence, $x_1^2 + x_2^2 + x_3^2 = c_0$ has no solution for $c_0 < 0$ and *P* has just two real zeros $\pm \sqrt{-c_0}$. The zero set of $P \in \mathbb {R}[t]$ over the split quaternions is always infinite.

We continue by considering monic polynomials whose constant coefficient is not real. Such a polynomial is given by *P* in (3.1) with $a = 1$, $b_1 = b_2 = b_3 = 0$ and $c \notin \mathbb {R}$.

#### Theorem 3.5

The polynomial $P = t^2 + c \in \mathbb {S}[t]$, where $c \in \mathbb {S}{\setminus }\mathbb {R}$, admits a factorization if and only if

- $${{\,\mathrm{Im}\,}}(c) \ {{{\,\mathrm{Im}\,}}(c)}^* > 0$$ or
- $$c {c}^* \ge 0$$ and $$c_0 < 0$$.

#### Proof

We solve the equation system (3.2) which, in the current setup, reads as

$$\begin{aligned} x_0^2 - x_1^2 + x_2^2 + x_3^2 + c_0&= 0, \\ 2 x_0 x_1 + c_1&= 0, \\ 2 x_0 x_2 + c_2&= 0, \\ 2 x_0 x_3 + c_3&= 0. \end{aligned}$$

The assumption $x_0 = 0$ implies $c_1 = c_2 = c_3 = 0$ and contradicts $c \notin \mathbb {R}$. Hence, we can plug $x_1 = -\frac{c_1}{2 x_0}$, $x_2 = -\frac{c_2}{2 x_0}$, $x_3 = -\frac{c_3}{2 x_0}$, in the first equation and obtain $\frac{1}{4 x_0^2} (4 x_0^4 + 4 c_0 x_0^2 - c_1^2 + c_2^2 + c_3^2) = 0$ with up to four distinct solutions

3.3 $$\begin{aligned} \begin{aligned} x_0 = \pm \frac{1}{\sqrt{2}}\sqrt{- c_0 \pm \sqrt{c_0^2 + c_1^2 - c_2^2 - c_3^2}}&= \pm \frac{1}{\sqrt{2}}\sqrt{- c_0 \pm \sqrt{c {c}^*}} \\&= \pm \frac{1}{\sqrt{2}}\sqrt{- c_0 \pm \sqrt{c_0^2 + {{\,\mathrm{Im}\,}}(c){{{\,\mathrm{Im}\,}}(c)}^*}} \end{aligned}\nonumber \\ \end{aligned}$$

over $\mathbb {C}$. We are only interested in real solutions and it is easy to see that all expressions in (3.3) yield non-real values if and only if $c {c}^* < 0$ or $c {c}^* \ge 0$, $c_0 > 0$ and ${{\,\mathrm{Im}\,}}(c) \ {{{\,\mathrm{Im}\,}}(c)}^* < 0$. We already verified $x_0 = 0$ to be invalid whence also the case ${{\,\mathrm{Im}\,}}(c) \ {{{\,\mathrm{Im}\,}}(c)}^* = 0$ and $c_0 \ge 0$ is excluded.

Hence, *P* has no split quaternion zeros and therefore does not admit a factorization if and only if

- $$c {c}^* < 0$$ or
- $$c {c}^* \ge 0$$, $$c_0 > 0$$ and $${{\,\mathrm{Im}\,}}(c) \ {{{\,\mathrm{Im}\,}}(c)}^* < 0$$ or
- $${{\,\mathrm{Im}\,}}(c) \ {{{\,\mathrm{Im}\,}}(c)}^* = 0$$ and $$c_0 \ge 0$$.

The negation of these conditions are easily shown to be equivalent to

- $${{\,\mathrm{Im}\,}}(c) \ {{{\,\mathrm{Im}\,}}(c)}^* > 0$$ or
- $$c {c}^* \ge 0$$ and $$c_0 < 0$$,

thus finishing the proof. $\square$

Still assuming that *P* is monic, we are left with the case where $b \notin \mathbb {R}$. Due to the assumed dependency of the coefficients there exist $\lambda$, $\mu \in \mathbb {R}$ such that $c = \lambda + \mu b$ and we can write $P = t^2 + b t + \lambda + \mu b$.

#### Theorem 3.6

Consider the split quaternion polynomial $P = t^2 + b t + \lambda + \mu b \in \mathbb {S}[t]$, where $b \in \mathbb {S}{\setminus }\mathbb {R}$, $b_0 = 0$ and $\lambda$, $\mu \in \mathbb {R}$.

- If $b {b}^* > 0$, then *P* admits a factorization.
- Provided that $b {b}^* = 0$, then *P* admits a factorization if and only if $\lambda + \mu ^2 = 0$ or $\lambda < 0$.
- Provided that $b {b}^* < 0$, then *P* admits a factorization if and only if $\lambda + \mu ^2 = 0$ or $b {b}^* + 4 \lambda \le 0$ and $b {b}^* + 4 \lambda \le 4 \mu \sqrt{- b {b}^*} \le -(b {b}^* + 4 \lambda )$.

#### Proof

First, let us assume that $b {b}^* > 0$. We pick a quadratic factor $M = t^2 + m_1 t + m_0 \in \mathbb {R}[t]$ of the norm polynomial $P {P}^*$ and compute the remainder polynomial $R = P - M = (b - m_1) t + \lambda + \mu b - m_0$ when dividing *P* by *M*. By the generic factorization algorithm, *P* admits a factorization if the leading coefficient of *R* is invertible. This is guaranteed by non-negativity of its norm $(b - m_1) {(b - m_1)}^* = b {b}^* + m_1^2 > 0$.

Next, we assume that $b {b}^* = 0$. If $\lambda + \mu ^2 = 0$, then obviously $P = (t + \mu ) (t - \mu + b)$ is a factorization. If $\lambda < 0$, then *P* factors as $P = (t - h_1) (t - h_2)$ where

$$\begin{aligned} h_{1,2} = \pm \sqrt{-\lambda } - \frac{1}{2} b \left( 1 \pm \frac{\mu }{\sqrt{-\lambda }}\right) . \end{aligned}$$

Conversely, if *P* admits a factorization, then *P* has a right zero. Such a zero is a solution of the equation system (3.2) which, in our case, reads as

3.4 $$\begin{aligned} \begin{aligned} x_0^2 - x_1^2 + x_2^2 + x_3^2 - b_1 x_1 + b_2 x_2 + b_3 x_3 + \lambda&= 0, \\ 2 x_0 x_1 + b_1 x_0 + b_3 x_2 - b_2 x_3 + \mu b_1&= 0, \\ 2 x_0 x_2 + b_2 x_0 + b_3 x_1 - b_1 x_3 + \mu b_2&= 0, \\ 2 x_0 x_3 + b_3 x_0 - b_2 x_1 + b_1 x_2 + \mu b_3&= 0. \end{aligned} \end{aligned}$$

Assuming that $x_0 \ne 0$, we can substitute $x_1 = -(b_1 (\mu + x_0))(2 x_0)^{-1}$, $x_2 = -(b_2 (\mu + x_0))(2 x_0)^{-1}$, $x_3 = -(b_3 (\mu + x_0))(2 x_0)^{-1}$ into the first equation and obtain $(4x_0^4 + (b {b}^* + 4\lambda ) x_0^2 - \mu ^2 b {b}^*)(4x_0^2)^{-1} = x_0^2 + \lambda = 0$. Since $x_0 \ne 0$, a real solution exists if and only if $\lambda < 0$. Considering solutions with $x_0 = 0$ the equations system (3.4) simplifies to

$$\begin{aligned} -x_1^2 + x_2^2 + x_3^2 - b_1 x_1 + b_2 x_2 + b_3 x_3 + \lambda&= 0, \\ b_3x_2 - b_2 x_3 + \mu b_1&= 0, \\ b_3x_1 - b_1 x_3 + \mu b_2&= 0, \\ -b_2x_1 + b_1 x_2 + \mu b_3&= 0. \end{aligned}$$

The conditions $b \notin \mathbb {R}$ and $b {b}^* = 0$ imply $b_1 \ne 0$ and the solution set of the equation system given by the last three equations is of dimension one. It can be parameterized by $\{ x_1 = \alpha ,\ x_2 = (b_2 \alpha - b_3 \mu )b_1^{-1},\ x_3 = (b_3 \alpha + b_2 \mu )b_1^{-1}:\alpha \in \mathbb {R}\}$. Substituting these solutions into the first equation yields $\lambda + \mu ^2 = 0$. This concludes the proof of the second statement.

Assuming that $b {b}^* < 0$, we can factor the norm polynomial as

$$\begin{aligned} P {P}^*= & {} (t^2 + \lambda )^2 + (t + \mu )^2 b {b}^*\\= & {} (t^2 + \lambda + \sqrt{- b {b}^*} (t + \mu )) (t^2 + \lambda - \sqrt{- b {b}^*} (t + \mu )). \end{aligned}$$

If $b {b}^* + 4 \lambda \le 0$ and $b {b}^* + 4 \lambda \le 4 \mu \sqrt{- b {b}^*} \le -(b {b}^* + 4 \lambda )$, then $P {P}^*$ has even real linear factors

$$\begin{aligned} P {P}^*&= L_1 L_2 L_3 L_4, \quad \text {where} \\ L_{1,2}&= t + \frac{1}{2} \left( \sqrt{- b {b}^*} \pm \sqrt{- (b {b}^* + 4 \lambda ) - 4 \sqrt{- b {b}^*} \mu } \right) , \\ L_{3,4}&= t - \frac{1}{2} \left( \sqrt{- b {b}^*} \pm \sqrt{- (b {b}^* + 4 \lambda ) + 4 \sqrt{- b {b}^*} \mu } \right) . \end{aligned}$$

Defining $M :=L_1 L_4$ and computing $R = r_1 t + r_0 = P - M \in \mathbb {S}[t]$ yields a remainder polynomial with leading coefficient

$$\begin{aligned} r_1 = b - \frac{1}{2} \left( \sqrt{- (b {b}^* + 4 \lambda ) + 4 \sqrt{- b {b}^*} \mu } + \sqrt{- (b {b}^* + 4 \lambda ) - 4 \sqrt{- b {b}^*} \mu } \right) . \end{aligned}$$

The polynomial *P* admits a factorization by means of the generic factorization algorithm if the norm $r_1 {r_1}^* = b {b}^* + \frac{1}{2} ( \sqrt{(b {b}^* + 4 \lambda )^2 + 16 \mu ^2 b {b}^*} - (b {b}^* + 4\lambda ) )$ of $r_1$ is different from zero. This is, indeed, the case as $b {b}^* \ne 0$ and $\mu ^2+\lambda \ne 0$. If $\mu ^2+\lambda = 0$, then *P* admits the factorization $P = (t + \mu ) (t - \mu + b)$ anyway. Similar to above considerations, a detailed inspection of the equation system (3.4) shows that no solutions exist if the conditions $\lambda + \mu ^2 = 0$ or $b {b}^* + 4 \lambda \le 0$ and $b {b}^* + 4 \lambda \le 4 \mu \sqrt{- b {b}^*} \le -(b {b}^* + 4 \lambda )$ are violated. $\square$

### Factorization of Monic Polynomials with Independent Coefficients

In [8] the authors showed that the polynomial *P* in (3.1) admits a factorization if its coefficients *a*, *b*, *c* are linearly independent and the leading coefficient *a* is invertible. Assuming, without loss of generality, that $a = 1$, we recall this result and provide an improved version of the second half of the proof in [8], namely the case where the general factorization algorithm is not applicable.

#### Theorem 3.7

([10]) The polynomial $P = t^2 + b t + c \in \mathbb {S}[t]$ admits a factorization if its coefficients 1, *b*, *c* are linearly independent.

#### Proof

Let $M_1 \in \mathbb {R}[t]$ be a monic quadratic factor of the norm polynomial $P {P}^*$ and compute the corresponding linear remainder polynomial $R_1 \in \mathbb {S}[t]$ such that $P = M_1 + R_1$. If $R_1 {R_1}^* \ne 0$, one can compute a factorization of *P* using the generic factorization algorithm. Hence, we continue by assuming that $R_1 {R_1}^* = 0$. Linear independence of the coefficients of *P* implies linear independence of the coefficients of $R_1$. Consequently, $R_1$ parameterizes a null line (Lemma 2.2). Consider the complementary monic quadratic factor $M_2 \in \mathbb {R}[t]$ of $P{P}^*$ defined by $P {P}^* = M_1 M_2$, and the corresponding remainder polynomial $R_2$ such that $P = M_2 + R_2$. From

$$\begin{aligned} \begin{aligned} P {P}^*&= (M_1 + R_1) {(M_1 + R_1)}^* \\&= M_1^2 + M_1 R_1 + M_1 {R_1}^* = M_1 (M_1 + R_1 + {R_1}^*) \end{aligned} \end{aligned}$$

we conclude that $M_2 = M_1 + R_1 + {R_1}^*$ and $R_2 = -{R_1}^*$. Hence, $R_2$ parameterizes a null line as well. The two null lines belong to different families of rulings of $\mathcal {N}$. Without loss of generality we assume that the null line parameterized by $R_1$ is a right ruling. Moreover, we can assume that the linear coefficient of $M_1$ is zero by applying a suitable parameter transformation ($t \mapsto t + {\tilde{m}}$ where ${\tilde{m}} \in \mathbb {R}$) to *P*.

Next we will show that $M_1 = t^2 + m \in \mathbb {R}[t]$ and $R_1 = r_1 t + r_0$ have a common right zero. By Corollary 2.7, there exists an $h \in \mathbb {S}$ such that $-r_0 = r_1 h$. Although Theorem 2.8 provides an explicit formula to compute such an $h \in \mathbb {S}$ in terms of $r_1$ and $r_0$, any $h \in \mathbb {S}$ fulfilling the relation $-r_0 = r_1 h$ will do and we choose one. Then the two-parametric set of right zeros of $R_1$ can be parameterized by $h + \lambda {r_1}^* + \mu {r_1}^* \mathbf {i}$ where $\lambda$, $\mu \in \mathbb {R}$. The norm of such an element reads as

$$\begin{aligned}&{(h + \lambda {r_1}^* + \mu {r_1}^* \mathbf {i})}^* (h + \lambda {r_1}^* + \mu {r_1}^* \mathbf {i}) \\&\quad = ({h}^* + \lambda r_1 - \mu \mathbf {i}r_1) (h + \lambda {r_1}^* + \mu {r_1}^* \mathbf {i}) \\&\quad = {h}^* h + \lambda {h}^* {r_1}^* + \mu {h}^* {r_1}^* \mathbf {i}+ \lambda r_1 h - \mu \mathbf {i}r_1 h\\&\quad = {h}^* h - \lambda {r_0}^* - \mu {r_0}^* \mathbf {i}- \lambda r_0 + \mu \mathbf {i}r_0 \\&\quad = {h}^* h - \lambda ({r_0}^* + r_0) - \mu ({r_0}^* \mathbf {i}- \mathbf {i}r_0). \end{aligned}$$

We choose $\lambda$ and $\mu$ such that this norm is equal to *m*, the constant coefficient of $M_1$, and in addition the real part of $h + \lambda {r_1}^* + \mu {r_1}^* \mathbf {i}$ is equal to zero. This is possible because the coefficient matrix

$$\begin{aligned} \begin{pmatrix} -r_0 - {r_0}^* &{} -{r_0}^*\mathbf {i}+ \mathbf {i}r_0 \\ {{\,\mathrm{Re}\,}}({r_1}^*) &{} {{\,\mathrm{Re}\,}}({r_1}^*\mathbf {i}) \end{pmatrix} \end{aligned}$$

of the underlying system of linear equations is singular precisely if the positive parts of $r_0$ and $r_1$ are linearly dependent. But then the null line spanned by $[r_0]$ and $[r_1]$ contains a point with zero positive part which is not possible. With above’s choice we have that $h + \lambda {r_1}^* + \mu {r_1}^* \mathbf {i}$ satisfies all conditions from Lemma 3.2. Hence, $h + \lambda {r_1}^* + \mu {r_1}^* \mathbf {i}$ is not only a right zero of $R_1$ but also a zero of $M_1$. Therefore, it is also a zero of *P* whence *P* admits a factorization. $\square$

The following example illustrates the “interesting” case in the proof of Theorem 3.7.

#### Example

Consider the polynomial $P = t^2 + (1 + \mathbf {k}) t + 2 + \mathbf {i}+ \mathbf {j}+ \mathbf {k}\in \mathbb {S}$. Its norm polynomial factors into $P {P}^* = M_1 M_2$ with $M_1 = t^2 + 1$ and $M_2 = t^2 + 2t + 3$. The respective remainder polynomials $R_1$, $R_2 \in \mathbb {S}$ such that $P = M_1 + R_1 = M_2 + R_2$ read as $R_1= (1 + \mathbf {k}) t + 1 + \mathbf {i}+ \mathbf {j}+ \mathbf {k}$ and $R_2 = (\mathbf {k}- 1) t - 1 + \mathbf {i}+ \mathbf {j}+ \mathbf {k}$. Both, $R_1$ and $R_2$ are null lines since $R_1 {R_1}^* = R_2 {R_2}^* = 0$, whereas only $R_1$ is a right ruling of $\mathcal {N}$. According to Theorem 2.8, the two-parametric set of right zeros of $R_1 = r_1 t + r_0 = (1 + \mathbf {k}) t + 1 + \mathbf {i}+ \mathbf {j}+ \mathbf {k}$ is parameterized by $h + \lambda {r_1}^* + \mu {r_1}^* \mathbf {i}$ with $\lambda$, $\mu \in \mathbb {R}$ and $h = - 1 - \mathbf {i}\in \mathbb {S}$. The conditions on the norm and the real part of $h + \lambda {r_1}^* + \mu {r_1}^* \mathbf {i}$ yield the two equations $\lambda - 1 = 0$ and $1 - 2 \lambda - 2 \mu = 0$ with the unique solution $\lambda = 1$ and $\mu = -\frac{1}{2}$. Indeed, the split quaternion $h + {r_1}^* - \frac{1}{2} {r_1}^* \mathbf {i}= -\frac{3}{2} \mathbf {i}+ \frac{1}{2} \mathbf {j}- \mathbf {k}$ is a zero of *P* and right division of *P* by $t + \frac{3}{2} \mathbf {i}- \frac{1}{2} \mathbf {j}+ \mathbf {k}$ yields the factorization $P = (t + 1 - \frac{3}{2} \mathbf {i}+ \frac{1}{2} \mathbf {j}) (t + \frac{3}{2} \mathbf {i}- \frac{1}{2} \mathbf {j}+ \mathbf {k})$.

Note that the two polynomials $M_1$ and $M_2$ are irreducible, hence there is no quadratic factor of the norm polynomial $P {P}^*$ yielding a non-null line as remainder polynomial and therefore the possibility to avoid above’s procedure.

### Geometric Interpretation for Factorizability of Monic Polynomials

Theorems 3.5 and 3.6 relate factorizability of a quadratic split quaternion polynomial to validity of certain inequalities. Some of these conditions are not very intuitive but necessary in order to cover all special cases by the algebraic approach. However, we can give an alternative characterization of factorizability by interpreting the factorization algorithm for quadratic split quaternions geometrically. It turns out that this alternative characterization covers the statement in Theorem 3.7 as well. Hence, the geometrical approach allows a unified characterization of factorizability for quadratic split quaternions with invertible leading coefficient without inconvenient case distinctions.

Consider a monic split quaternion polynomial $P = t^2 + b t + c \in \mathbb {S}[t]$ and a monic real polynomial $M \in \mathbb {R}[t]$, both of degree two. Let $t_1$, $t_2 \in \mathbb {C}$ be the two roots of $M = (t - t_1)(t - t_2)$. Denote by $R = P - M$ the remainder polynomial of *P* divided by *M*. Because of $M(t_1) = M(t_2) = 0$ we have

$$\begin{aligned} P(t_1) = M(t_1) + R(t_1) = R(t_1) \quad \text {and}\quad P(t_2) = M(t_2) + R(t_2) = R(t_2) \end{aligned}$$

and, provided that $t_1 \ne t_2$, the remainder *R* is the unique interpolation polynomial with respect to the interpolation data set $\{ (t_1, P(t_1)),$ $(t_2, P(t_2)) \}$. Hence *R* parameterizes the straight line $[P(t_1)] \vee [P(t_2)]$ or, if these two points coincide, the point $[P(t_1)] = [P(t_2)]$.

If $t_1 = t_2$ and thus $P(t_1) = P(t_2)$, the linear interpolation polynomial is not well defined. Instead, the remainder polynomial *R* describes the tangent of the rational curve parameterized by *P* at the point $[P(t_1)]$. In order to see this, we compute

$$\begin{aligned} P(t_1) + P'(t_1) (t - t_1) = t_1^2 + b t_1 + c + (2 t_1 + b)(t - t_1) = (2 t_1 + b ) t + c - t_1^2. \end{aligned}$$

It is equal to the remainder polynomial

$$\begin{aligned} R = P - M = t^2 + b t + c - (t - t_1)(t - t_1) = (2 t_1 + b) t + c - t_1^2. \end{aligned}$$

Note that $P'(t_1)$ might vanish, that is, $b = -2 t_1$. In this case, the parametric representation of the tangent as well as the remainder polynomial are constant and equal to $c-t_1^2$.

In the context of the generic factorization algorithm the real polynomial *M* is one of the quadratic factors of the norm polynomial $P {P}^*$ and $t_1$, $t_2 \in \mathbb {C}$ are parameter values where the rational curve parameterized by *P* intersects the null quadric $\mathcal {N}$. Hence, the remainder polynomial *R* parameterizes the line $[P(t_1)] \vee [P(t_2)]$ spanned by these two intersection points provided $[P(t_1)] \ne [P(t_2)]$. If these points are equal and $t_1 \ne t_2$, it is a linear parametrization of the single point $[P(t_1)] = [P(t_2)]$. If, finally $t_1 = t_2$ (and hence also $P(t_1) = P(t_2)$), *R* parameterizes the tangent of the rational curve *P* in $[P(t_1)]$ (or again a single point if $P'(t_1) = 0$).

#### Definition 3.8

Consider a monic split quaternion polynomial $P = t^2 + b t + c \in \mathbb {S}[t]$ of degree two. Let $t_1$, $t_2$, $t_3$, $t_4 \in \mathbb {C}$ be the four roots of the norm polynomial $P {P}^* \in \mathbb {R}[t]$. We define the (at most six) *remainder polynomials* of *P* by $R_{ij} :=P - M_{ij}\in \mathbb {S}[t]$ where $M_{ij} :=(t- t_i)(t-t_j) \in \mathbb {R}[t]$ for *i*, $j \in \{ 1,2,3,4 \}$ and $i < j$.

Note that we only consider remainder polynomials that have *real* split quaternion coefficients, that is, we only use quadratic factors $M_{ij} \in \mathbb {R}[t]$. The curve parameterized by *P* intersects the null quadric $\mathcal {N}$ in four points $[P(t_1)]$, $[P(t_2)]$, $[P(t_3)]$, $[P(t_4)] \in \mathbb {P}(\mathbb {S})$. Their respective parameter values $t_1$, $t_2$, $t_3$, $t_4 \in \mathbb {C}$ are the four roots of the norm polynomial $P {P}^*$. Hence, the polynomials $M_{ij} \in \mathbb {R}[t]$ are the real quadratic factors of $P {P}^*$ and the remainder polynomials $R_{ij} \in \mathbb {S}[t]$ are the interpolation polynomials with respect to the interpolation data sets $\{ (t_i, P(t_i)),$ $(t_j, P(t_j)) \}$. The interpolation polynomials are defined in above’s sense, i.e. they can be constant or, if $t_i = t_j$, may parameterize the tangent of the curve at the point $[P(t_i)]$.

#### Lemma 3.9

Let $P = t^2 + b t + c \in \mathbb {S}[t]$ be a split quaternion polynomial and $R = r_1 t + r_0 \in \mathbb {S}[t]$ be one of its remainder polynomials. If *R* is of degree one, then *R* has a either a unique root, *R* parameterizes a null line or $r_1$ and $r_0$ are linearly dependent.

#### Proof

If $r_1 {r_1}^* \ne 0$, then $r_1^{-1} r_0 \in \mathbb {S}$ is the unique root of *R*. Hence, we assume that $r_1 {r_1}^* = 0$. Moreover, we assume that $r_0$ and $r_1$ are linearly independent, that is, *R* parameterizes a straight line $\ell$ in $\mathbb {P}(\mathbb {S})$. In order to show that $\ell$ is a null line, we show that there are at least three intersection points between $\mathcal {N}$ and $\ell$. One of them is $[r_1] = [R(\infty )] :=[\lim _{t\rightarrow \infty }t^{-\deg {P}}P(t)]$.

Let $M = (t-t_1)(t-t_2) \in \mathbb {R}[t]$ be the quadratic factor of $P {P}^*$ such that $P = M + R$. Further intersection points of $\mathcal {N}$ and $\ell$ are $[R(t_1)] = [P(t_1)]$ and $[R(t_2)] = [P(t_2)]$. Thus we have found three different intersection points unless $[R(t_1)] = [R(t_2)]$. Because *R* is a linear polynomial with independent coefficients, equality of $[R(t_1)]$ and $[R(t_2)]$ implies $t_1 = t_2$ and $[P(t_1)] = [R(t_1)] = [R(t_2)] = [P(t_2)]$. As shown at the beginning of this subsection, $\deg R = 1$ and independence of $r_1$ and $r_0$ implies that this point is a regular point of the rational curve *P* and $\ell$ is its tangent. We conclude that $\ell$ is also tangent to $\mathcal {N}$ in $[R(t_1)] = [R(t_2)]$. Since it also intersects $\mathcal {N}$ in one further point $[r_1]$, it is a null line. $\square$

#### Remark 3.10

If *P* has a real linear factor $t - r \in \mathbb {R}[t]$, then $M = (t-r)^2$ is a quadratic factor of $P{P}^*$ and $t - r$ is a linear factor of the corresponding remainder polynomial *R*. In this case, the coefficients $r_1$ and $r_0$ in Lemma 3.9 are linearly dependent. Conversely, linear dependency of $r_1$ and $r_0$ in Lemma 3.9 is equivalent to *R* having a real root $r \in \mathbb {R}$. If *r* is also a root of *M*, then *P* has the real factor $t - r \in \mathbb {R}[t]$. In this case, factorizability is obvious whence we exclude it in the following.

#### Theorem 3.11

The polynomial $P = t^2 + b t + c \in \mathbb {S}[t]$ without a real factor admits a factorization if and only if there is a polynomial $R \in \mathbb {S}[t]$ of degree one with linearly independent coefficients among the set of its remainder polynomials.

#### Proof

Without loss of generality we assume that the remainder polynomial $R_{12}$ has degree one and its coefficients are linearly independent. If the leading coefficient of $R_{12}$ is invertible, $R_{12}$ has a unique root and then the generic factorization algorithm yields a factorization of *P*. If the leading coefficient is not invertible then $R_{12}$ parameterizes a null line by Lemma 3.9. Provided that $R_{12}$ parameterizes a right ruling of $\mathcal {N}$, then we have seen in the proof of Theorem 3.7 that *P* admits a factorization. If $R_{12}$ parameterizes a left ruling of $\mathcal {N}$, the complementary remainder polynomial $R_{34}$ parameterizes, again by the proof of Theorem 3.7, a right ruling and *P* once more admits a factorization.

Conversely assume that *P* admits a factorization, that is $P = (t-h_1) (t-h_2)$ with $h_1$, $h_2 \in \mathbb {S}$. Then

$$\begin{aligned} \begin{aligned} P {P}^*&= (t-h_1) (t-h_2) {((t-h_1) (t-h_2))}^* \\&= (t-h_1) {(t-h_1)}^* (t-h_2) {(t-h_2)}^* \end{aligned} \end{aligned}$$

and $M = (t-h_2) {(t-h_2)}^*$ is a quadratic factor of the norm polynomial $P {P}^*$. We compute the according remainder polynomial $R \in \mathbb {S}[t]$ such that $P = M + R$. Since $t-h_2$ is a right factor of $M = {(t-h_2)}^* (t-h_2)$, it is also a right factor of $R = P - M$. Hence, there exists a split quaternion $r \in \mathbb {S}$ such that $R = r (t - h_2)$. If $r = 0$, we have $P = M$ which contradicts the assumption that *P* has no real factor. Thus, *R* has degree one. In order to show independence of its coefficients we assume the opposite, i.e. there exists a real number $\alpha \in \mathbb {R}$ such that $0 = \alpha r - r h_2 = r (\alpha - h_2)$. Obviously $\alpha$ is a root of *R* and $[\alpha - h_2] \in \mathcal {N}$, that is $0 = (\alpha - h_2) {(\alpha - h_2)}^* = M(\alpha )$. Consequently, $P(\alpha ) = M(\alpha ) + R(\alpha ) = 0$ and *P* has the real factor $t-\alpha$ by Lemma 3.1. $\square$

#### Remark 3.12

In our proof of Theorem 3.11 we appeal to the proof of Theorem 3.7 whose assumptions are slightly different. This is admissible: The assumed independence of coefficients in Theorem 3.7 implies independence of the coefficients of the remainder polynomial *R*. In Theorem 3.11, this is not a conclusion but an assumption.

The considerations on remainder polynomials above allow to translate the condition in Theorem 3.11, that there be a remainder polynomial of degree one, to the possibility to find an interpolation data set $\{ (t_i, P(t_i)),$ $(t_j, P(t_j)) \}$ such that the according interpolation polynomial parameterizes a real line. This is not possible precisely if each interpolation polynomial parameterizes a point or a non-real line and yields a profound geometrical interpretation of the equality and inequality conditions in Theorems 3.5 and 3.6, respectively. It also clarifies the cause of non-factorizability in these theorems.

Although the norm polynomial $P {P}^*$ might have four distinct roots $t_1$, $t_2$, $t_3$, $t_4 \in \mathbb {C}$, the line segment parameterized by *P* in Theorems 3.5 or 3.6 intersects $\mathcal {N}$ only at two distinct (not necessarily real) points. Hence, two of the four points represented by $P(t_1)$, $P(t_2)$, $P(t_3)$ and $P(t_4)$ coincide, respectively. We set $P_\ell :=P(t_\ell )$ for $\ell \in \{1,2,3,4\}$ and, without loss of generality, assume $[P_1] = [P_3]$ and $[P_2] = [P_4]$. The coefficients of $P_1$ or $P_2$ might be non-real. If so, the points $[P_1]$ and $[P_2]$ are complex conjugates, respectively, in the sense of [3, Section 5.1]. At any rate, these two points are also the intersection points of $\mathcal {N}$ and the line segment’s support line, that is the line $[1] \vee [c]$ in Theorem 3.5 or the line $[1] \vee [b]$ in Theorem 3.6.

#### Geometric Interpretation of Theorem 3.5

The curve parameterized by *P* is a half-line with start-point *c* and direction 1 in pseudo-Euclidean space $\mathbb {S}$ and one of the two projective line segments with endpoints [1] and [*c*] in $\mathbb {P}(\mathbb {S})$. All factorizability conditions of Theorem 3.5 pertain to *c* but their geometric interpretation indirectly also depends on the projective point $[1] \in \mathbb {P}(\mathbb {S})$ because of assumptions we made “without loss of generality” (in particular monicity of *P*).

- The sign of $c{c}^*$ distinguishes between points of the null quadric $\mathcal {N}$, its exterior, and its interior. The condition $c{c}^* \ge 0$ means, for example that the end point [*c*] is on $\mathcal {N}$ or in the interior of $\mathcal {N}$.
- The sign of ${{\,\mathrm{Im}\,}}(c){{{\,\mathrm{Im}\,}}(c)}^*$ distinguishes between points of the “asymptotic” cone with vertex [1] over the intersection of $\mathcal {N}$ with the “ideal” plane $c_0 = 0$, its exterior, and its interior. For example, points satisfying $c{c}^* \ge 0$ and ${{\,\mathrm{Im}\,}}(c){{{\,\mathrm{Im}\,}}(c)}^* < 0$ lie inside the null quadric and outside its asymptotic cone.
- If ${{\,\mathrm{Im}\,}}(c){{{\,\mathrm{Im}\,}}(c)}^* > 0$, then the points $[P_1]$ and $[P_2]$ are complex conjugates. The real line connecting them can be parameterized by the interpolation polynomial with respects to the data set $\{ (t_1,P_1),$ $(t_2,P_2) \}$ since $t_1$ and $t_2$ can be chosen as complex conjugates from the set $\{ t_1,t_2,t_3,t_4 \}$.
- The two endpoints separate the line $[1] \vee [c]$ into two line segments. The sign of $c_0$ determines which segment is parameterized by *P*, where $c_0 < 0$ denotes the one intersecting the ideal plane.
- If ${{\,\mathrm{Im}\,}}(c){{{\,\mathrm{Im}\,}}(c)}^* \le 0$, then the points $[P_1]$ and $[P_2]$ are real. If in addition $c {c}^* < 0$, then either $t_1$ and $t_2$ or $t_3$ and $t_4$ are non-real. Hence, none of the interpolation polynomials parameterize a real line: Those involving the pairs $(t_1, t_3)$, $(t_1, t_4)$, $(t_2, t_3)$ or $(t_2, t_4)$ parameterize non-real lines and those involving the pairs $(t_1,t_2)$ or $(t_3,t_4)$ are constant.
- If ${{\,\mathrm{Im}\,}}(c){{{\,\mathrm{Im}\,}}(c)}^* \le 0$ and $c {c}^* \ge 0$ the sign of $c_0$ is crucial. Similar to the item above, $c_0 \ge 0$ yields only interpolation polynomials parameterizing a non-real line or a point. Conversely, $c_0 < 0$ implies that $t_1$, $t_2$, $t_3$ and $t_4$ are real and we can find interpolation polynomials parameterizing a real line, e.g. the one according to the data set $\{ (t_1,P_1),$ $(t_2,P_2) \}$.

With exception of the sign of $c_0$, all inequality conditions are of projective nature. In the affine space $\mathbb {S}$, the sign of $c_0$ distinguishes between half-spaces. A natural framework for a unified geometric interpretation of all inequalities is *oriented projective geometry* [7, 14].

#### Geometric Interpretation of Theorem 3.6

Again, the polynomial *P* parameterizes a projective line segment or possibly a projective line in $\mathbb {P}(\mathbb {S})$. The endpoints of the line segment are $[e_{1,2}] = [\pm 2 \mu \sqrt{\lambda + \mu ^2} + 2 (\lambda + \mu ^2) \mp b \sqrt{\lambda + \mu ^2}]$. At any rate, the segment contains the point [1].

- Similar as in Theorem 3.5, the sign of $b{b}^* = {{\,\mathrm{Im}\,}}(b) {{{\,\mathrm{Im}\,}}(b)}^*$ distinguishes between lines or line segments with supporting line that lies on the null quadric’s asymptotic cone or in the cone’s interior/exterior.
- The case $b {b}^* > 0$ is identical to the case ${{\,\mathrm{Im}\,}}(c) \ {{{\,\mathrm{Im}\,}}(c)}^* > 0$ in Theorem 3.5.
- If $b {b}^* = 0$ or $b {b}^* < 0$, then $[P_1]$ and $[P_2]$ are real. The respective conditions $\lambda < 0$ or $b {b}^* + 4 \lambda \le 0$ and $b {b}^* + 4 \lambda \le 4 \mu \sqrt{- b {b}^*} \le -(b {b}^* + 4 \lambda )$ ensures that $t_1$, $t_2$, $t_3$ and $t_4$ are real as well whence there exists an interpolation polynomial parameterizing a real line. Otherwise, the parameter values $t_1$, $t_2$, $t_3$, $t_4$ are non-real and all interpolation polynomials parameterize non-real lines or points.
- Because 1 and *b* are linearly independent, the condition $\lambda + \mu ^2 = 0$ is necessary and sufficient for the existence of a real zero of *P*. This is equivalent to *P* being a linear parametrization of the line $[1] \vee [b]$ multiplied with a linear real polynomial. This is a trivial case which we have excluded.

Based on these considerations we can state a simple geometric criterion for the existence of factorizations in case of monic polynomials with dependent coefficients. This covers all polynomials $P = at^2 + bt + c$ in (3.1) where *a*, *b* and *c* are linearly dependent and the leading coefficient *a* is invertible since multiplication with $a^{-1}$ yields a monic polynomial. Moreover, if *a*, *b* and *c* are linearly dependent so are 1, $a^{-1}b$ and $a^{-1}c$ and vice versa. In fact we even covered those cases where the leading coefficient *a* of *P* is not invertible but the curve parameterized by *P* is not contained in the null quadric $\mathcal {N}$, i.e. the norm polynomial does not vanish. As long as there is a point on the curve which is not contained in $\mathcal {N}$, one can apply a proper parameter transformation to *P* such that the leading coefficient becomes invertible. Factorizability of the thus obtained polynomial guarantees factorizability of the initial one.

##### Theorem 3.13

Assume that the polynomial $P \in \mathbb {S}[t]$ is of degree two, has linearly dependent coefficients, no real factor of positive degree, and a non-vanishing norm polynomial. Denote by *L* the vector sub-space (of dimension two) spanned by the coefficients of *P*. There exists a factorization of *P* if and only if the point sets $\{[P(t)] \mid t \in \mathbb {R}\cup \{\infty \}\}$ (line segment parameterized by *P*) and [*L*] intersect $\mathcal {N}$ in the same number of points.

**Fig. 1:**
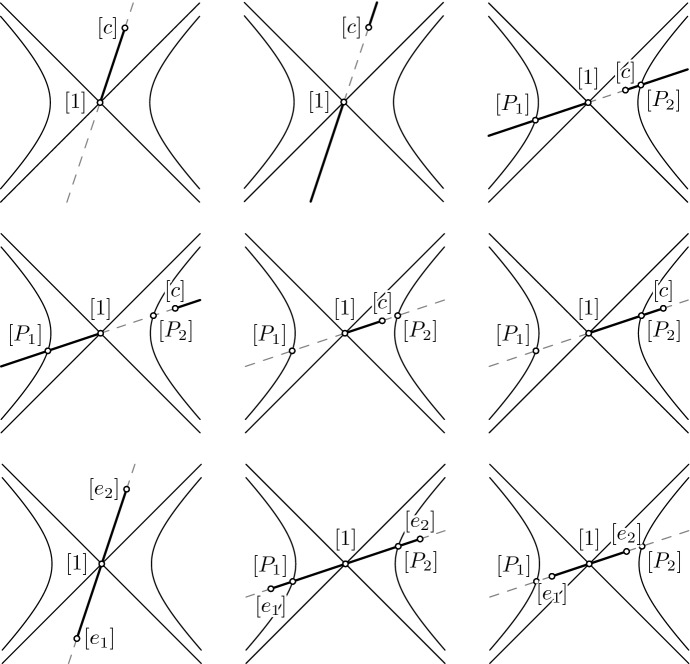
Geometric interpretation of factorizability in case of dependent coefficients

The content of Theorem 3.13 is visualized in Fig. 1. Images in the first and second row refer to the geometric interpretation of Theorem 3.5, the last row refers to Theorem 3.6. Images in the first row and the first and second image in the last row correspond to cases that admit factorizations. All other images correspond to cases that don’t.

#### Geometric Interpretation of Theorem 3.7

In this, the coefficients of *P* are independent whence it parameterizes a (regular) conic section ${\mathcal {C}}$ in $\mathbb {P}(\mathbb {S})$. It intersects the null quadric $\mathcal {N}$ in four points, not necessarily real or distinct. Nonetheless, a suitable choice of a remainder polynomial (which corresponds to a suitable choice of a line) is always possible: We may connect a generic pair of distinct real intersection points, a pair of conjugate complex intersection points or pick the tangent in a real intersection point of multiplicity at least two. The most interesting case is that of ${\mathcal {C}}$ lying in a tangent plane of $\mathcal {N}$. In this case, the intersection of ${\mathcal {C}}$ and $\mathcal {N}$ will always contain a left and a right ruling. In the proof of Theorem 3.7 we have shown that right ruling is always a suitable choice.

**Fig. 2:**
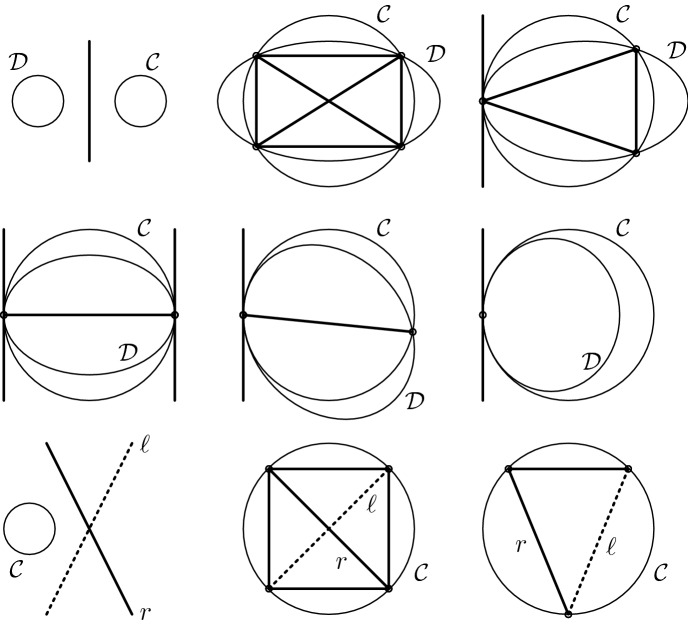
Geometric interpretation of Theorem 3.7

This is illustrated in Fig. 2. In the first and second row, we assume that ${\mathcal {C}}$ is not in a tangent plane of $\mathcal {N}$ so that we actually look at the intersection of two regular conics, ${\mathcal {C}}$ and the intersection conic ${\mathcal {D}}$ of $\mathcal {N}$ with the plane of ${\mathcal {C}}$. For diverse relative positions of ${\mathcal {C}}$ and ${\mathcal {D}}$, suitable connecting lines are drawn in bold line style. In the top-left image, all intersection points are non-real but a real connecting line does exist.

The bottom row illustrates cases where ${\mathcal {C}}$ is in a tangent plane of $\mathcal {N}$. Thus, the plane of ${\mathcal {C}}$ intersects $\mathcal {N}$ in a left ruling $\ell$ and a right ruling *r*. Once again, suitable choices of lines are drawn in bold and potentially invalid lines in dotted line style.

### Factorization of Polynomials with Non-invertible Leading Coefficient

So far, we considered polynomials $P = at^2 + bt + c$ in (3.1) with invertible leading coefficient and non-vanishing norm polynomial. Taking into account the already explained possibility of re-parameterization just before Theorem 3.13, the only missing case in our discussion so far is that the curve parameterized by *P* is contained in $\mathcal {N}$. This is the case if and only if the norm polynomial vanishes:

3.5 $$\begin{aligned} P {P}^* = a {a}^* t^4 + (a {b}^* + b {a}^*) t^3 + (a {c}^* + c {a}^* + b {b}^*) t^2 + (b {c}^* + c {b}^*) t + c {c}^* = 0.\nonumber \\ \end{aligned}$$

It will turn out that factorizations always exist. In our investigation, we distinguish two cases:

- *a*, *b*, $c \in \mathbb {S}$ linearly dependent $\leadsto$ Theorem 3.14
- *a*, *b*, $c \in \mathbb {S}$ linearly independent $\leadsto$ Theorem 3.16

We start the discussion by assuming that the coefficients *a*, *b* and *c* are linearly dependent. Hence, the corresponding points [*a*], [*b*] and [*c*] lie on a line. Since the norm polynomial vanishes this line is a ruling of the null quadric $\mathcal {N}$.

#### Theorem 3.14

The polynomial $P = a t^2 + b t + c \in \mathbb {S}[t]$ with $P {P}^* = 0$ and linearly dependent coefficients admits a factorization.

In the ensuing proof of Theorem 3.14 it is possible that the coefficients *b* or $c \in \mathbb {S}$ vanish whence the points [*b*] and $[c] \in \mathbb {P}(\mathbb {S})$ become undefined. For the sake of readability, we do not always take into account this possibility in our proof which, nonetheless, is also valid for these special cases.

#### Proof of Theorem 3.14

Without loss of generality, we may assume that [*a*], [*b*] and [*c*] lie on a right ruling since *P* is factorizable if and only if its conjugate ${P}^* = {a}^* t^2 + {b}^* t + {c}^*$ is factorizable and conjugation exchanges right rulings and left rulings. The proof is subdivided into four different cases.

For the first two cases, we assume that $b = 0$. If *c* is a real multiple of *a*, which is equivalent to $[a] = [c]$, then *P* can be written as $P = a {\hat{P}}$ with ${\hat{P}} \in \mathbb {R}[t]$. Since ${\hat{P}}$ admits a factorization in terms of Lemma 3.2, so does *P*.

If $[a] \ne [c]$ then $[a] \vee [c]$ is a right ruling of $\mathcal {N}$. Due to Theorem 2.8 there exists a split quaternion *h* such that $c = a h$. We write $P = a (t^2 + h)$ and show that $t^2 + h$ admits a factorization. There are two degrees of freedom in the choice of *h*, namely the two real parameters $\lambda$ and $\mu$ of Theorem 2.8. We set them both equal to zero. Then $h = h_0 + h_1 \mathbf {i}$ is an element of the sub-ring $\langle 1, \mathbf {i}\rangle _\mathbb {R}\subset \mathbb {S}$. The assumption $[a] \ne [c]$ yields $h_1 \ne 0$. We have $h {h}^* = h_0^2 + h_1^2 > 0$ and ${{\,\mathrm{Im}\,}}(h) {{{\,\mathrm{Im}\,}}(h)}^* = h_1^2 > 0$. Hence, $t^2 + h$ is factorizable by means of Theorem 3.5.

We are left with the case that $b \ne 0$ and [*a*], [*b*], [*c*] lie on a right ruling of $\mathcal {N}$.

If $[a] = [b]$ we may write $b = \alpha a$ with $\alpha \in \mathbb {R}\setminus \{0\}$. Moreover, by Theorem 2.8, there exist $h = h_0 + h_1\mathbf {i}$ such that $c = ah$ whence $P = a(t^2 + \alpha t + h)$. By a suitable parameter transformation, we can eliminate the coefficient of *t* in $t^2 + \alpha t + h$ while preserving the vector part ${{\,\mathrm{Im}\,}}(h)$ of the constant coefficient. Existence of a factorization is once more guaranteed by Theorem 3.5.

If $[a ] \ne [b]$, we chose, again according to Theorem 2.8, $h = h_0 + h_1 \mathbf {i}$ such that $b = a h$. Moreover, there exist $\alpha , \beta \in \mathbb {R}$ such that $c = \alpha a + \beta b$ due to linear dependency of *a*, *b* and *c*. We can write $P = a (t^2 + h t + \alpha + \beta h)$ and since $h {h}^* > 0$, the polynomial $t^2 + h t + \alpha + \beta h$ fulfills the condition of Theorem 3.6 to be factorizable. $\square$

#### Remark 3.15

In the proof of Theorem 3.14, we set the two real parameters $\lambda$ and $\mu$ equal to zero. But there are infinitely many possible choices for these parameters according to Theorem 2.8. The crucial ingredient in the proof is that the norm of *h* or ${{\,\mathrm{Im}\,}}h$ is strictly positive. Since these norms depend continuously on $\lambda$ and $\mu$, strict positivity is preserved for infinitely many choices of $\lambda$ and $\mu$. Hence, there are infinitely many factorizations for the second, third and fourth case. In the first case ($b = 0$, $[a] = [c]$) existence of infinitely many factorizations is guaranteed by Corollary 3.3.

Finally, we present the missing factorization result for split quaternion polynomials with vanishing norm polynomial and independent coefficients.

#### Theorem 3.16

The polynomial $P = a t^2 + b t + c \in \mathbb {S}[t]$ with $P {P}^* = 0$, $a \ne 0$ and linearly independent coefficients admits a factorization.

#### Proof

The condition $P {P}^* = 0$ implies that each coefficient in Eq. (3.5) vanishes. In particular, we have

3.6 $$\begin{aligned} \begin{aligned} 0&= b {a}^* + a {b}^* = 2 \, q(b,a) = 2 \, q({b}^*,{a}^*) = {b}^* a + {a}^* b,\\ 0&= b {c}^* + c {b}^* = 2 \, q(b,c) = 2 \, q({b}^*,{c}^*) = {b}^* c + {c}^* b. \end{aligned} \end{aligned}$$

If *b* is not invertible, i.e. $b {b}^* = 0$, Eq. (3.6) implies that not only the points [*a*], [*b*], $[c] \in \mathbb {P}(\mathbb {S})$ are contained in $\mathcal {N}$, but also the lines $[a] \vee [b]$, $[b] \vee [c]$. This is not possible because *P* parameterizes a non-singular planar section of $\mathcal {N}$.

Hence *b* is invertible and the split quaternion $h :=-b^{-1} c$ is a zero of *P*:

$$\begin{aligned} P(h)&= a h^2 + b h + c = a (- b^{-1} c)^2 + b (- b^{-1} c) + c \\&= \frac{1}{(b {b}^*)^2} a {b}^* c {b}^* c \overset{(3.6)}{=} - \frac{1}{(b {b}^*)^2} a {b}^* \underbrace{c {c}^*}_{=0} b = 0. \end{aligned}$$

By Lemma 3.1, $t - h$ is a right factor of *P* and a factorization exists. $\square$

Theorem 3.16 in combination with Theorems 3.7 or 3.14 implies a corollary each.

#### Corollary 3.17

Any quadratic split quaternion polynomial with linearly independent coefficients admits a factorization.

#### Corollary 3.18

Any quadratic split quaternion polynomial with vanishing norm admits a factorization.

## Future Research

We have presented a complete discussion of factorizability of quadratic polynomials over the split quaternions and provided a geometric interpretation in the (oriented) projective space over the split quaternions. A natural next step is, of course, factorizability questions for higher degree polynomials. We expect to be able to re-use ideas and techniques of this paper. One thing that is already clear is existence of non-factorizable polynomials of arbitrary degree.

Other questions of interest include factorization results for different algebras. One obstacle to generalizations is the lack of a suitable substitute of quaternion conjugation, that is, a linear map that gives inverse elements up to scalar multiples. Existence of such a map and its exploitation for factorization on suitable and interesting sub-algebras are on our research agenda as well. Preliminary results in Conformal Geometric Algebra already exist.
